# Supplementary material for: Enhancement of Textural and Sensory Characteristics of Wheat Bread Using a Chickpea Sourdough Fermented with a Selected Autochthonous Microorganism
Source: Foods. 2023 Aug 18;12(16):3112. doi: 10.3390/foods12163112 (PMC10453481; doi:10.3390/foods12163112)
Supplement: Supplementary file 1 [file foods-12-03112-s001.zip › foods-2556552-supplementary.pdf]

Table S1. Addition of different carbohydrates in fermented chickpea flour before drying and abbreviations used in the text.

Description	Abbreviation after freeze-drying	Abbreviation after oven drying
Liquid Sourdough	FDS	ODS
100g Liquid Sourdough and 15g Maltodextrin	FDSM	ODSM
100g Liquid Sourdough and 15g Trehalose	FDST	ODST
100g Liquid Sourdough and 15g Maltodextrin and 5g Trehalose	FDSDM+T	ODSDM+T
100g Liquid Sourdough and 15g Sucrose	FDSS	ODSS
100g Liquid Sourdough and 15g Maltodextrin and 5g Sucrose	FDSDM+S	ODSDM+S

Table S2: Bread attributes for sensory analysis.

Parameter	Descriptor	Definition	
**Aroma**	Wheat Type	Basic wheat aroma	1 (lack) - 9 (too intense)
	Non-Wheat Type	Aroma different from wheat (not-identified)	
	Butter-like	Aroma associated with ‘butter–like’ note	
**Taste**	Wheat Type	Basic wheat flavor	
	Non-Wheat Type	Flavor different from wheat (non-identified)	
	Sour	Basic sour flavor	
	Bitter	Basic bitter flavor	
	Butter-like	Sour and cheesy, ‘buttery-like’	
**Perception of bread staling after 4 Days of storage**		In comparison with 1st Day control bread (CB)	1 (fresh) – 9 (‘staled’ bread, rejected by the panelists)
**Butyric aroma after 4 Days of storage**	Butter-like	Aroma associated with ‘butter–like’ note	1 (lack) - 9 (too intense)

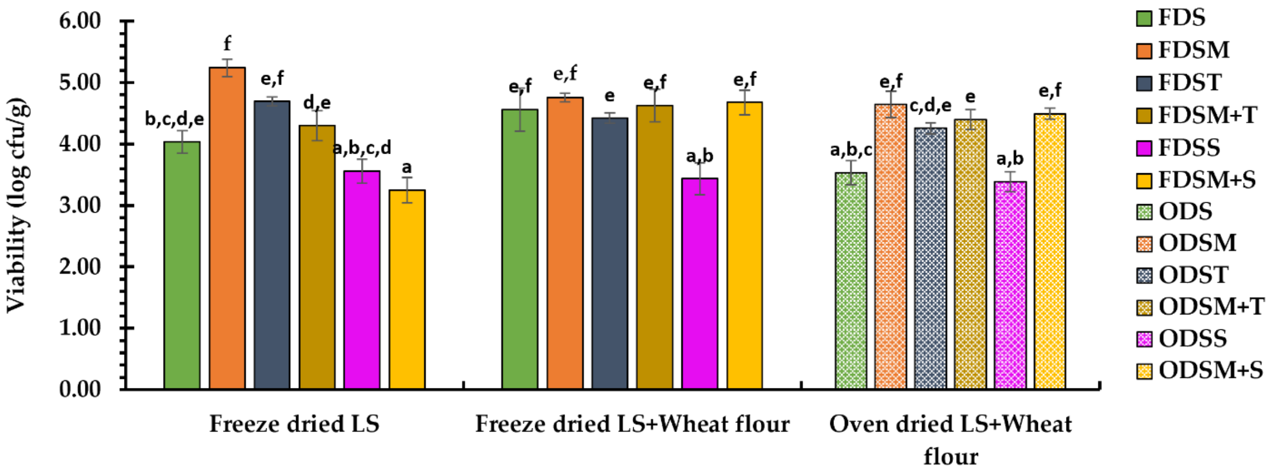

Figure S1. Viability of *C. perfringens* CP8 in dried chickpea sourdoughs, following drying of liquid sourdough (LS) with two different methods (freeze-drying, FDS, and oven-drying, ODS) with or without further addition of wheat flour and with the addition of several carbohydrates, sucrose (S), trehalose (T), maltodextrin (M) in several combinations; mean values with different letter indicate significant differences ($p < 0.05$). Notation of samples as in Table S1.
